# Animal model studies on viral infections

**DOI:** 10.3389/fmicb.2014.00672

**Published:** 2014-12-03

**Authors:** Akio Adachi, Tomoyuki Miura

**Affiliations:** ^1^Department of Microbiology, Institute of Health Biosciences, The University of Tokushima Graduate SchoolTokushima, Japan; ^2^Laboratory of Primate Model, Experimental Research Center for Infectious Diseases, Institute for Virus Research, Kyoto UniversityKyoto, Japan

**Keywords:** animal models, viral infections, viral replication, viral pathogenicity, anti-viral strategies

One of the major missions of animal virology is to understand how viruses replicate and cause asymptomatic/symptomatic conditions in individuals (Nomaguchi and Adachi, 2010). It is especially important for virologists who work on viruses pathogenic for humans to elucidate bases underlying the *in vivo* viral characteristics. Toward this end, animal model studies in some ways are necessary to precisely analyze the *in vivo* situation, and also are essential for developing countermeasures against virus infections. Since a full variety of viruses with distinct biological properties exist, we virologists should study “the target virus” in a specialized manner, in addition to common theoretical/experimental approaches. The Research Topic entitled “Animal model studies on viral infections” collects articles that describe the studies on numerous virus species for their animal models, or those at various stages toward animal experiments.

Articles in this Research Topic were written by experts in various research fields, and can be fairly grouped into a few categories: (i) descriptions/evaluations/new challenges of animal model studies for investigating the biology of viruses; (ii) experimental materials/methods for upcoming animal model studies; (iii) observations important for animal model studies. (i) Reynaud and Horvat (2013) have described the animal models for human herpesvirus 6 to better understand its pathogenic property. Studies on filoviruses, classified as biosafety level-4 and represent a serious world-wide problem today, have been reviewed by Nakayama and Saijo (2013). Mailly et al. (2013) have focused on the quest for appropriate animal models for hepatitis C virus. Clark et al. (2013) have discussed about the use of non-human primates as models for dengue hemorrhagic fever/dengue shock syndrome. Ohsugi (2013) has summarized mouse strains transgenic for the *tax* gene of human T-cell leukemia virus type 1 (HTLV-1). Also, a bovine model for HTLV-1 pathogenesis has been described by Aida et al. (2013). Challenging new attempts to establish human immunodeficiency virus type 1 (HIV-1)/macaque infection models have been reviewed by Misra et al. (2013), and also by Saito and Akari (2013). Another approach to understand HIV-1 biology *in vivo* has been described by Matsuyama-Murata et al. (2013). (ii) Kodama et al. (2013) has described a new and simple method to prepare human dendritic cells from peripheral blood mononuclear cells. Doi et al. (2013) have summarized their studies on macaque-tropic HIV-1 clones. Ikeno et al. (2013) has reported a new, sensitive, and quantitative system to monitor measles virus infection in humanized mice. Iwami et al. (2013) have summarized the quantification of viral infection dynamics based on various quantitative analyses. (iii) Tada et al. (2013) have suggested that LEDGF/p75 may be a cellular factor acting as a species-barrier against HIV-1 in mouse cells. Kuwata et al. (2013) have shown that simian immunodeficiency virus may acquire the increased infectivity and resistance to neutralizing antibodies by truncation of its gp41 cytoplasmic tail. Ohsugi et al. (2013) have reported that natural infection status of laboratory mice by murine norovirus. Finally, Kajitani et al. (2013) have described the possible involvement of E1^∧^E4 protein of human papillomavirus type 18 in its differentiation-dependent life cycle.

We are proud to add our “Animal model studies on viral infections” to a series of Research Topic in Frontiers in Microbiology. A wide variety of DNA and RNA viruses are covered by this special issue consisting of original research, review, mini-review, methods, and opinion articles. As we described in the beginning, animal studies are certainly required for understanding virus replicative/pathogenic properties *in vivo* and for overcoming virally-caused infectious diseases. We human virologists should make every effort to fight against numbers of unique pathogenic viruses.

## Conflict of interest statement

The authors declare that the research was conducted in the absence of any commercial or financial relationships that could be construed as a potential conflict of interest.

## References

[B1] Aida Y. Murakami H. Takagashi M. Takeshima S. (2013). Mechanisms of pathogenesis induced by bovine leukemia virus as a model for huma T-cell leukemia virus. Front. Microbiol. 4:328. 10.3389/fmicb.2013.00328 24265629 PMC3820957

[B2] Clark K. Onlamoon N. Hsiao H.-M. Perng G. Villinger F. (2013). Can non-huma primates serve as models for investigating dengue disease pathogenesis? Front. Microbiol. 4:305. 10.3389/fmicb.2013.00305 24130557 PMC3795305

[B3] Doi N. Okubo A. Yamane M. Sakai Y. Adachi A. Nomaguchi M. (2013). Growth potentials of CCR5-tropic/CXCR4-tropic HIV-1mt clones in macaque cells. Front. Microbiol. 4:2188. 10.3389/fmicb.2013.00218 23908651 PMC3725405

[B4] Ikeno S. Suzuki M. Muhsen M. Ishige M. Kobayashi M. Ohno S.. (2013). Sensitive detection of measles virus infection in the blood and tissues of humanized mouse by one-step quantitative RT-PCR. Front. Microbiol. 4:298. 10.3389/fmicb.2013.00298 24130556 PMC3795360

[B5] Iwami S. Koizumi Y. Ikeda H. Kakizoe Y. (2013). Quantification of viral infection dynamics in animal experiments. Front. Microbiol. 4:264. 10.3389/fmicb.2013.002264 24058361 PMC3767920

[B6] Kajitani N. Satsuka A. Yoshida S. Sakai K. (2013). HPV 18 E1^∧^E4 is assembled into aggresome-like compartment and involved in sequestration of viral oncoproteins. Front. Microbiol. 4:251. 10.3389/fmicb.2013.00251 23986755 PMC3754431

[B7] Kodama A. Tanaka R. Saito M. Ansari A. Tanaka Y. (2013). A novel and simple method for generation of human dendritic cells from unfractionated peripheral blood mononuclear cells within 2 days: its application for induction of HIV-1-reactive CD4^+^ T cells in the hu-PBL SCID mice. Front. Microbiol. 4:292. 10.3389/fmicb.2013.00292 24098298 PMC3784773

[B8] Kuwata T. Takaki K. Enomoto I. Yoshimura K. Matsushita S. (2013). Increased infectivity in human cells and resistance to antibody-mediated neutralization by truncation of the SIV gp41 cytoplasmic tail. Front. Microbiol. 4:117. 10.3389/fmicb.2013.00117 23717307 PMC3653066

[B9] Mailly L. Robinet E. Meuleman P. Baumert T. F. Zeisel M. B. (2013). Hepatitis C virus infection and related liver disease: the quest for the best animal model. Front. Microbiol. 4:212. 10.3389/fmicb.2013.00212 23898329 PMC3724122

[B10] Matsuyama-Murata M. Inaba K. Horiuchi R. Fukazawa Y. Ibuki K. Hayami M.. (2013). Genetic similarity of circulating and small intestinal virus at the end stage of acute pathogenic simian-human immunodeficiency virus infection. Front. Microbiol. 4:204. 10.3389/fmicb.2013.00204 23885255 PMC3717482

[B11] Misra A. Thippeshappa R. Kimata J. T. (2013). Macaques as model hosts for studies of HIV-1 infection. Front. Microbiol. 4:176. 10.3389/fmicb.2013.00176 23825473 PMC3695370

[B12] Nakayama E. Saijo M. (2013). Animal models for Ebola and Marburg virus infections. Front. Microbiol. 4:267. 10.3389/fmicb.2013.00267 24046765 PMC3763195

[B13] Nomaguchi M. Adachi A. (2010). Virology as biosystematics: towards understanding the viral infection biology. Front. Microbiol. 1:2. 10.3389/fmicb.2010.00002 21747778 PMC3128384

[B14] Ohsugi T. (2013). A transgenic mouse model of huma T cell leukemia virus type 1-associated diseases. Front. Microbiol. 4:49. 10.3389/fmicb.2013.00049 23483782 PMC3592262

[B15] Ohsugi T. Matsuura K. Kawabe S. Nakamura N. Kumar J. M. Wakamiya M.. (2013). Natural infection of murine norovirus in conventional and specific pathogen-free laboratory mice. Front. Microbiol. 4:12. 10.3389/fmicb.2013.00012 23386847 PMC3558705

[B16] Reynaud J. Horvat B. (2013). Animal models for human herpesvirus 6 infection. Front. Microbiol. 4:174. 10.3389/fmicb.2013.00174 23847599 PMC3701164

[B17] Saito A. Akari H. (2013). Macaque-tropic human immunodeficiency virus type 1: breaking out of the host restriction factors. Front. Microbiol. 4:187. 10.3389/fmicb.2013.00187 23847610 PMC3705164

[B18] Tada T. Kadoki M. Yang L. Tokunaga K. Iwakura Y. (2013). Transgenic expression of the human LEDGF/p75 gene relieves the species barrier against HIV-1 infection in mouse cells. Front. Microbiol. 4:377. 10.3389/fmicb.2013.00377 24381568 PMC3865800

